# Cervicovaginal microbiome alterations across HPV infection

**DOI:** 10.3389/fcimb.2026.1907605

**Published:** 2026-09-21

**Authors:** Laura Chulenbayeva, Aidana Rakhmankulova, Nazira Kamzayeva, Samat Kozhakhmetov, Milan Terzic, Gauri Bapayeva, Gulzhanat Aimagambetova, Yevgeniy Kim, Berik Primbetov, Balkenzhe Imankulova, Kuralay Kongrtay, Nazira Kadroldinova, Makhabbat Galym, Sanimkul Makhambetova, Kadisha Nurgaliyeva, Zhanar Abdiyeva, Zhanar Zhumakanova, Talshyn Ukybassova, Almagul Kushugulova

**Affiliations:** 1 Laboratory of Microbiome, Center for Life Sciences, National Laboratory Astana, Nazarbayev University, Astana, Kazakhstan; 2 Department of Biomedical Sciences, School of Medicine, Nazarbayev University, Astana, Kazakhstan; 3 Clinical Academic Department of Women’s Health, Corporate Fund “University Medical Center”, Astana, Kazakhstan; 4 Department of Surgery, School of Medicine, Nazarbayev University, Astana, Kazakhstan; 5 Department of Normal Physiology, Sechenov First Moscow State Medical University, Moscow, Russia

**Keywords:** cervical dysbiosis, cervicovaginal microbiome, community state type, human papillomavirus, shotgun metagenomics

## Abstract

**Introduction:**

The cervicovaginal microbiome is a proposed modifier of HPV-associated cervical disease, yet its relationship with viral type heterogeneity, cytological grade, and community state type (CST) remains incompletely understood.

**Methods:**

This cross-sectional study characterized cervicovaginal microbiome composition and functional potential across HPV infection status, viral type categories (No HPV, HPV High Risk, HPV16, HPV18, HPV Other), cervical cytological grades, and CSTs in 311 non-pregnant women using whole-genome shotgun metagenomic sequencing, integrated diversity analyses, MaAsLin2 differential abundance testing, and HUMAnN 3.0 functional pathway profiling.

**Results:**

HPV-positive samples showed increased bacterial species richness, and a reciprocal *Lactobacillus*-to-*Gardnerella* dominance shift compared to HPV-negative samples. HPV Other showed the highest bacterial species richness and distinct taxonomic and predicted functional associations, including higher inferred abundance of siderophore- and lipopolysaccharide-biosynthesis pathways; however, overall species-level community composition did not differ significantly across HPV type groups. CST I and CST II exhibited compositional stability regardless of HPV status, while CST IV showed pronounced *Lactobacillus* depletion in HPV-positive women. LSIL was associated with lower *Fannyhessea vaginae* and *Alloscardovia omnicolens* abundance and higher *Phocaeicola vulgatus* abundance relative to NILM.

**Discussion:**

These findings indicate that oncogenic potential and dysbiosis severity are not aligned, and that CST type modifies HPV–microbiome interactions.

## 1 Introduction

Human papillomavirus (HPV) infection remains the primary etiological factor in cervical carcinogenesis, with high-risk HPV types responsible for virtually all cervical cancer cases worldwide (Walboomers et al., 1999; Okunade, 2020). However, the transition from transient HPV infection to persistent infection and subsequent progression to cervical intraepithelial neoplasia and invasive carcinoma occurs in only a fraction of infected individuals, suggesting that host factors beyond viral oncogenicity play critical roles in disease pathogenesis (Schiffman and Wentzensen, 2013). The cervicovaginal microbiome has emerged as a potential modifier of HPV-associated disease risk, with accumulating evidence indicating that bacterial community composition influences viral persistence, immune responses, and epithelial transformation (Mitra et al., 2016; Kyrgiou et al., 2017; Łaniewski et al., 2020).

The cervicovaginal microbiome exhibits distinct community state types (CSTs), primarily classified by dominant *Lactobacillus* species or absence thereof (Ravel et al., 2011). Lactobacillus-dominant communities, particularly those dominated by L. crispatus (CST I), are associated with vaginal health through production of lactic acid, hydrogen peroxide, and bacteriocins that maintain acidic pH and inhibit pathogen colonization (O’Hanlon et al., 2013; Petrova et al., 2015; Amabebe and Anumba, 2018). Conversely, polymicrobial communities depleted of *Lactobacillus* and enriched in anaerobic bacteria (CST IV), characteristic of bacterial vaginosis, associate with increased susceptibility to sexually transmitted infections, including HPV, and adverse reproductive outcomes (Ma et al., 2012; Borgdorff et al., 2014). However, the specific interactions between HPV infection, viral type heterogeneity, cervical cytological changes, and microbiome composition remain incompletely characterized.

Important knowledge gaps remain regarding whether HPV–microbiome associations differ by viral type group, whether different HPV categories are associated with distinct bacterial profiles, and how cervicovaginal microbiome composition differs across cytological grades. Furthermore, the context-dependency of HPV-microbiome interactions across different baseline CST configurations requires systematic investigation. Previous studies have yielded conflicting results regarding Lactobacillus depletion in HPV-positive women, with some reporting significant associations while others found minimal differences, potentially reflecting heterogeneity in HPV types, cytological grades, or CST distributions (Di Paola et al., 2017; Tamarelle et al., 2019; Norenhag et al., 2020).

The present study addresses these gaps through comprehensive characterization of cervicovaginal microbiome composition across HPV status, HPV type categories (including separate analysis of low-risk HPV Other, high-risk HPV types, HPV16, and HPV18), cervical cytological grades (NILM, ASC-US, ASC-H, LSIL), and baseline CST types. We employed whole-genome shotgun metagenomic sequencing, combined with alpha and beta diversity analyses, differential abundance testing, and functional pathway profiling, to characterize HPV type-specific microbiome signatures, identify grade-associated bacterial markers, and determine CST-dependent responses to HPV infection.

## 2 Materials and methods

### 2.1 Study design and population

This cross-sectional study was conducted at the National Research Center for Mother and Child Health, a tertiary academic medical center in Astana, Kazakhstan. Non-pregnant women aged 18–46 years with regular menstrual cycles and an intact cervix were recruited from the outpatient gynecology clinic. Participants were excluded for major chronic or immunocompromising conditions, acute inflammation, recent antibiotic/probiotic/immunosuppressive therapy, smoking, intrauterine device use, prior HPV vaccination, or recent invasive gynecological procedures. Samples with incomplete metadata, insufficient sequencing data, or missing CST classification were excluded from the relevant analyses. A subset of the HPV-negative, NILM (normal cytology) participants characterized here (n = 92) was previously reported as a dedicated multi-kingdom baseline cohort in Rakhmankulova et al. (2026), which used the same BioProject accession (PRJNA1337009) to describe bacterial, viral, fungal, and archaeal community structure in HPV-negative women prior to HPV-positive comparison. The present study extends those baseline findings by incorporating HPV-positive comparator groups (HPV16, HPV18, HPV Other, HPV High Risk), cervical cytological grade stratification, and CST-based differential abundance and functional pathway analyses not covered in the earlier report.

### 2.2 Sample collection, DNA extraction and sequencing

Cervical samples were stored at −80 °C until processing. Total genomic DNA was extracted using the ZymoBIOMICS DNA Miniprep Kit according to the manufacturer’s protocol. Sterile nuclease-free water was included as a negative extraction control, and the ZymoBIOMICS Microbial Community Standard was used as a positive control. DNA samples were required to contain at least 1.0 μg total DNA, with good integrity and no visible impurities. DNA concentration was measured using a microplate reader with a broad-range DNA assay, and DNA integrity was assessed by agarose gel electrophoresis.

Raw paired-end sequencing reads were processed using fastp for adapter trimming and quality filtering. Read pairs were discarded when adapter contamination was detected, when more than 5 bp contained ambiguous nucleotides, or when more than 40% of bases were of low quality. Sliding-window trimming was performed using a 4-bp window and a Q15 threshold. The mean numbers of raw and clean reads were 48.5 million and 47.9 million reads per sample, respectively. Mean Q20/Q30 values were 98.2%/93.8% for raw reads and 99.0%/95.3% for clean reads.

Human-read depletion was performed by alignment against the human GRCh38 reference genome using Bowtie2 v2.4.4. In the original pipeline, read pairs failing concordant alignment to the human reference were retained using the --un-conc-gz option while preserving paired-read structure for downstream metagenomic classification. Residual host content after depletion was evaluated using Kraken2 v2.1.3. On average, 92.1% of reads in the Bowtie2-retained fraction were subsequently classified as Homo sapiens, indicating substantial residual host sequence after this permissive depletion step. Approximately 7.9% of reads in the retained fraction were not classified as human and comprised microbial and unclassified reads available for downstream taxonomic classification.

Taxonomic classification was performed using Kraken2 v2.1.3 against the PlusPFP Kraken2/Bracken database. Bracken v3.0 was subsequently used to refine genus- and species-level abundance estimates. Functional profiling was performed using HUMAnN 3.0 with UniRef90 protein annotations and MetaCyc pathway reconstruction. No predefined minimum non-host sequencing-depth threshold was applied in the original pipeline. Because effective bacterial sequencing depth varied substantially among samples, classified bacterial depth was quantified directly from the unnormalized Kraken2/Bracken bacterial count table and was explicitly evaluated in subsequent depth-corrected and sensitivity analyses. Classified bacterial sequencing depth was defined as the total number of reads assigned to the bacterial domain (d:Bacteria) in the unnormalized Kraken2/Bracken count table.

### 2.3 Groupings

Samples were analyzed according to four main clinical and microbiome grouping variables: HPV status, HPV type group, cytology/histology grade, and bacterial community state type (CST). HPV status was assessed using the RealBest DNA HPV High Carcinogenic Risk Genotype real-time PCR kit (Cat. No. C8899; Vector-Best), according to the manufacturer’s instructions. The assay detects the following high-risk HPV genotypes: 16, 18, 31, 33, 35, 39, 45, 51, 52, 56, 58, and 59. A sample was considered positive when at least one HPV genotype produced a cycle-threshold value of Ct ≤35. Samples without detection of any target genotype were classified as negative for the high-risk HPV types included in the assay. Whole-genome shotgun sequencing data were also screened for HPV sequences to identify genotypes not included in the PCR panel. The standard Reference sequence databases were used. Samples positive by either PCR or WGS were classified as HPV-positive. For descriptive analyses, HPV16 and HPV18 were retained as separate categories, other oncogenic genotypes were classified as HPV High Risk, and detected genotypes outside the predefined high-risk list were classified as HPV Other. The HPV Other group comprised samples in which WGS screening identified only genotypes classified as low-risk or non-carcinogenic by IARC (HPV6b, 7, 11, 26, 30, 32, 34, 53, 54, 71, 85, 90, 144, 156, and 166). A small number of HPV Other samples additionally showed trace-level WGS reads mapping to the high-risk genotypes HPV16, HPV31, or HPV35; because these reads represented less than 5% of the relative HPV sequence abundance within the sample, well below the threshold used to call a genotype present, these samples were retained in the HPV Other group rather than reclassified as HPV16, HPV High Risk, or HPV-positive for the corresponding high-risk type. Cytology/histology grade was categorized as NILM, ASC-US, LSIL, and ASC-H. CSTs were assigned manually from species-level relative abundance profiles following the five-community framework of Ravel et al. (2011). The dominant taxon was defined as the CST-associated bacterial species with the highest relative abundance within each sample. Samples were classified as CST I, CST II, CST III, or CST V when *Lactobacillus crispatus*, *L. gasseri*, *L. iners*, or *L. jensenii*, respectively, showed the highest relative abundance. Samples lacking dominance by these Lactobacillus species and characterized by predominance of anaerobic bacteria were assigned to CST IV. No fixed abundance threshold was used.

### 2.4 Statistical and bioinformatic analysis

Bacterial alpha- and beta-diversity analyses were performed using species-level bacterial relative-abundance features only. Observed richness was defined as the number of detected bacterial species per sample; Shannon diversity and Pielou evenness were calculated from the same species-level matrix.

Samples with zero total microbial abundance were excluded before diversity analysis. Because alpha-diversity and pathway-abundance measures were not assumed to follow a normal distribution, non-parametric tests were used for group comparisons. Differences across more than two groups were assessed using the Kruskal-Wallis test, whereas two-group and stratified HPV-positive versus HPV-negative comparisons were assessed using the Mann-Whitney U test.

Beta diversity was calculated using Bray-Curtis dissimilarity and visualized by principal coordinates analysis. Differences in overall community composition were tested using PERMANOVA with 999 permutations. Pseudo-F statistics, permutation p-values, and pseudo-R^2^ values were reported, with R^2^ interpreted as the proportion of total variation in the distance matrix attributable to the grouping variable. PERMDISP with 999 permutations was performed to assess homogeneity of within-group multivariate dispersion and to determine whether PERMANOVA findings could be influenced by differences in dispersion rather than group-centroid separation.

Because observed richness is sensitive to sequencing depth, we performed a depth-corrected sensitivity analysis using exact rarefaction of terminal species-level bacterial counts. Species counts summed exactly to total classified bacterial counts for each sample. All 311 samples were rarefied without replacement to 357,456 bacterial reads per sample, corresponding to the minimum classified bacterial depth. Rarefaction was repeated independently 100 times to assess sensitivity to stochastic subsampling. We additionally evaluated the association of observed richness with HPV status using linear regression adjusted for log10-transformed classified bacterial depth.

Species-level differential abundance was evaluated using MaAsLin2 in R. Two abundance datasets were used according to the primary predictor. CST-associated analyses were performed using a taxonomically collapsed species table to reduce artificial inflation from strain- and isolate-level labels. Assignments corresponding to Gardnerella vaginalis strains and reference isolates were aggregated into a single G. vaginalis feature, whereas the remaining unresolved Gardnerella assignments were combined as Gardnerella unspecified. Strain-like Lactobacillus assignments were aggregated to the corresponding species-level labels. HPV type group- and cytological grade-associated analyses were performed using the uncollapsed species table to retain finer species-, strain-, and isolate-level resolution. Taxon names were standardized for formatting, and only exact duplicate labels generated by formatting standardization were aggregated. No biological consolidation of Gardnerella, Lactobacillus, or other taxa was applied in the HPV type group and Grade models. Predefined implausible assignments, including Clostridium tetani, were excluded from both datasets. Taxon filtering was performed independently for the collapsed and uncollapsed datasets after matching participants with complete model covariates. Taxa were retained when detected in at least 10% of samples and when their mean relative abundance was at least 0.05%. Samples with zero total bacterial abundance or incomplete model covariates were excluded from the corresponding analysis. Because the input tables contained relative-abundance percentages, no additional normalization was applied within MaAsLin2. Relative abundances were log-transformed, and linear models were fitted without predictor standardization. Each model included HPV type group, CST group, cytological grade, age, number of deliveries, bacterial sequencing depth, and marital status as fixed effects. Age and number of deliveries were entered as mean-centered continuous covariates. Bacterial sequencing depth was calculated from the unnormalized Kraken2/Bracken bacterial count table, transformed as log10(depth + 1), and mean-centered. Marital status was modelled as a categorical variable comprising married, not married, and divorced/widowed categories. No HPV, CST I, NILM, and married status were used as the respective reference categories. Benjamini–Hochberg false-discovery-rate correction was applied, and q-values <0.05 were considered statistically significant. MaAsLin2 coefficients represent the direction and magnitude of association with log-transformed relative abundance relative to the relevant reference category while holding the remaining model variables constant. For visualization, up to six species with the lowest adjusted q-values for each primary predictor were displayed as boxplots. The boxplots show raw relative-abundance distributions, whereas the reported coefficients and q-values were obtained from the adjusted models. Coefficient heatmaps included all significant taxa up to a maximum of 50 features. Heatmap colors represent unscaled MaAsLin2 coefficients; rows were hierarchically clustered, whereas comparison columns were retained in predefined order.

To evaluate the robustness of differential-abundance results, MaAsLin2 analyses were additionally repeated after exclusion of samples below the 25th percentile of classified bacterial depth (3,108,006 reads). This retained 226 of 302 complete-case participants. The same feature matrices and model specifications as in the primary analyses were used, and taxa were not re-filtered after exclusion so that removal of low-depth samples was the only analytical change.

Categorical variables were compared using Pearson’s chi-squared test when expected cell counts were adequate. Fisher’s exact test was used for sparse contingency tables. For larger-than-2 × 2 tables where exact computation was impractical, Fisher’s exact test with Monte Carlo simulation was applied.

An additional exploratory HPV-positive versus HPV-negative taxonomic comparison was performed using Welch’s two-sample t-test, which does not assume equal group variances, together with Cohen’s d as a standardized effect-size measure. Before differential testing, the same predefined taxonomic exclusion filter used in the main bacterial analyses was applied to remove implausible environmental, animal-associated, and likely misclassified assignments at both genus and species levels. Taxa absent from both HPV groups were excluded. Positive Cohen’s d values indicated higher abundance in HPV-positive samples, whereas negative values indicated higher abundance in HPV-negative samples. Taxa meeting the exploratory thresholds of p < 0.05 and |Cohen’s d> 0.25 were included in the cladogram and effect-size plot. This analysis was considered exploratory, and nominal p-values were used for feature selection.

All microbiome, functional, and network analyses were performed in Python v3.12 using NumPy (v2.0.1) (Harris et al., 2020), SciPy (v1.15.1) (Virtanen et al., 2020), Pandas (The pandas development team, 2026), statsmodels (v0.14.4), scikit-learn (v1.6.1), Matplotlib (v3.10.0) (Hunter, 2007), seaborn (v0.13.2) (Waskom, 2021) and in R using MaAsLin2, ggplot2, pheatmap, dplyr, tidyr, and related packages. Demographic and baseline clinical characteristics were analyzed using Stata v18 (StataCorp, College Station, TX, USA).

## 3 Results

### 3.1 Clinical and demographic characteristics of the study population

A total of 311 non-pregnant women were included in the study. Of these, 148 (47.6%) were HPV-positive and 163 (52.4%) were HPV-negative. By HPV type group, 163 participants (52.4%) had no HPV infection, 64 (20.6%) were classified as HPV High Risk, 43 (13.8%) as HPV16, 34 (10.9%) as HPV Other (low-risk types), and 7 (2.3%) as HPV18. The mean age of participants was 34.5 ± 6.3 years and differed significantly across HPV type groups (p = 0.002), with HPV16 (31.4 ± 5.3 years) and HPV18 (30.4 ± 6.1 years) groups being younger compared to No HPV (35.3 ± 6.1 years) and HPV Other (35.3 ± 6.7 years) groups. Body mass index (mean 23.5 ± 4.3 kg/m^2^) and age at menarche (mean 13.4 ± 1.4 years) did not differ significantly across groups (p = 0.41 and p = 0.35, respectively). Because the HPV18 group comprised only 7 participants (2.3% of the cohort), demographic and clinical contrasts involving this group, including the younger age and lower parity noted above, are based on a small subgroup and should be regarded as descriptive and exploratory rather than definitive. Cytological grade distribution differed significantly across HPV type groups (p < 0.001). Overall, NILM was the most prevalent grade (142 women, 45.7%), followed by LSIL (79, 25.4%), ASC-US (73, 23.5%), and ASC-H (10, 3.2%). Among No HPV participants, NILM predominated (61.3%), whereas LSIL was the most common grade in HPV High Risk (43.8%) and HPV16 (55.8%) groups. The HPV Other group showed the highest proportion of NILM (67.6%). ASC-H was most prevalent in the HPV16 group (11.6%) and was rare in No HPV (0.6%). Community state type (CST) distribution did not differ significantly across HPV type groups (p = 0.074). CST IV was the most common overall (140 participants, 45.0%), followed by CST III (70, 22.5%), CST I (67, 21.5%), CST II (25, 8.0%), and CST V (9, 2.9%). The HPV Other and HPV16 groups had the highest proportions of CST IV (61.8% and 62.8%, respectively), while CST I was most prevalent in HPV High Risk (26.6%).

Regarding demographic and reproductive characteristics, the majority of participants identified as Kazakh (91.3%), with no significant difference in ethnicity across groups (p = 0.93). Most participants held a bachelor’s degree (69.8%), and educational level did not differ across HPV groups (p = 0.52). Marital status differed significantly (p = 0.008): married women comprised 65.6% of the total cohort and were most prevalent in the No HPV group (74.8%), while the proportions of unmarried and divorced/widowed women were higher among HPV-positive groups, particularly HPV18 (57.1% unmarried) and HPV Other (26.5% unmarried). The mean number of deliveries differed significantly across groups (p = 0.005), being lowest in the HPV18 group (0.5 ± 0.8) and highest in the No HPV group (1.9 ± 1.4). Mean cycle length also differed modestly across groups (p = 0.025), with HPV Other showing the longest mean cycle (34.3 ± 18.8 days) compared with HPV High Risk (28.8 ± 2.1 days). Menstrual cycle regularity (92.6% regular overall), menstrual phase at sampling, and days since last menstrual period did not differ significantly across groups (p = 0.69, p = 0.89, and p = 0.98, respectively). Full demographic and clinical characteristics stratified by HPV type group are presented in **Table 1**.

**Table 1:** Demographic and clinical characteristics of the study population stratified by HPV type group.

Variable	Total (n = 311)	HPV high risk (n = 64)	HPV other (n = 34)	HPV16 (n = 43)	HPV18 (n = 7)	No HPV (n = 163)	P-value	Test
HPV Status								
*Negative*	163 (52.4%)	0 (0.0%)	0 (0.0%)	0 (0.0%)	0 (0.0%)	163 (100.0%)		
*Positive*	148 (47.6%)	64 (100.0%)	34 (100.0%)	43 (100.0%)	7 (100.0%)	0 (0.0%)	<0.001	χ^2^
Community State Type (CST)								
*CST I (L. crispatus-dominant)*	67 (21.5%)	17 (26.6%)	5 (14.7%)	6 (14.0%)	1 (14.3%)	38 (23.3%)		
*CST II (L. gasseri-dominant)*	25 (8.0%)	3 (4.7%)	2 (5.9%)	5 (11.6%)	0 (0.0%)	15 (9.2%)		
*CST III (L. iners-dominant)*	70 (22.5%)	21 (32.8%)	4 (11.8%)	4 (9.3%)	1 (14.3%)	40 (24.5%)		
*CST IV (anaerobe-rich)*	140 (45.0%)	22 (34.4%)	21 (61.8%)	27 (62.8%)	5 (71.4%)	65 (39.9%)		
*CST V (L. jensenii-dominant)*	9 (2.9%)	1 (1.6%)	2 (5.9%)	1 (2.3%)	0 (0.0%)	5 (3.1%)	0.074	χ^2^
Cytological grade								
*NILM*	142 (45.7%)	11 (17.2%)	23 (67.6%)	6 (14.0%)	2 (28.6%)	100 (61.3%)		
*ASCUS*	73 (23.5%)	19 (29.7%)	6 (17.6%)	8 (18.6%)	1 (14.3%)	39 (23.9%)		
*LSIL*	79 (25.4%)	28 (43.8%)	4 (11.8%)	24 (55.8%)	3 (42.9%)	20 (12.3%)		
*ASC-H*	10 (3.2%)	4 (6.2%)	0 (0.0%)	5 (11.6%)	0 (0.0%)	1 (0.6%)	<0.001	χ^2^
Age (years), mean (SD)								
	34.5 (6.3)	34.9 (6.8)	35.3 (6.7)	31.4 (5.3)	30.4 (6.1)	35.3 (6.1)	0.002	ANOVA
BMI (kg/m^2^), mean (SD)								
	23.5 (4.3)	23.8 (3.8)	23.8 (4.8)	22.3 (4.5)	22.6 (4.0)	23.6 (4.3)	0.41	ANOVA
Age at menarche (years), mean (SD)								
	13.4 (1.4)	13.4 (1.4)	13.8 (2.0)	13.2 (1.1)	12.9 (0.7)	13.4 (1.2)	0.35	ANOVA
Cycle length (days), mean (SD)								
	30.0 (8.4)	28.8 (2.1)	34.3 (18.8)	28.9 (2.8)	28.3 (3.5)	29.9 (7.5)	0.025	ANOVA
Number of deliveries, mean (SD)								
	1.6 (1.4)	1.3 (1.3)	1.8 (1.5)	1.3 (1.2)	0.5 (0.8)	1.9 (1.4)	0.005	ANOVA
Cycle regularity								
*Regular*	288 (92.6%)	60 (93.8%)	29 (85.3%)	41 (95.3%)	7 (100.0%)	151 (92.6%)		
*Irregular*	22 (7.1%)	4 (6.2%)	4 (11.8%)	2 (4.7%)	0 (0.0%)	12 (7.4%)	0.69	χ^2^
Days since LMP at sampling, mean (SD)								
	75.7 (361.2)	90.7 (451.5)	54.8 (144.6)	69.0 (151.0)	15.6 (7.9)	78.4 (398.3)	0.98	ANOVA
Menstrual Phase at Sampling								
*Menstrual (0–5)*	23 (7.4%)	5 (7.8%)	1 (2.9%)	3 (7.0%)	1 (14.3%)	13 (8.0%)		
*Early follicular (6–10)*	59 (19.0%)	12 (18.8%)	9 (26.5%)	11 (25.6%)	1 (14.3%)	26 (16.0%)		
*Late follicular/ovulatory (11–16)*	66 (21.2%)	11 (17.2%)	8 (23.5%)	10 (23.3%)	2 (28.6%)	35 (21.5%)		
*Luteal (17–28)*	99 (31.8%)	23 (35.9%)	9 (26.5%)	8 (18.6%)	2 (28.6%)	57 (35.0%)		
*Delayed/prolonged (>28)*	64 (20.6%)	13 (20.3%)	7 (20.6%)	11 (25.6%)	1 (14.3%)	32 (19.6%)	0.89	χ^2^
Ethnicity								
*Kazakh*	284 (91.3%)	59 (92.2%)	29 (85.3%)	40 (93.0%)	7 (100.0%)	149 (91.4%)		
*Russian*	15 (4.8%)	3 (4.7%)	3 (8.8%)	1 (2.3%)	0 (0.0%)	8 (4.9%)		
*Other*	12 (3.9%)	2 (3.1%)	2 (5.9%)	2 (4.7%)	0 (0.0%)	6 (3.7%)	0.93	χ^2^
Education								
*Bachelor's degree*	217 (69.8%)	51 (79.7%)	21 (61.8%)	27 (62.8%)	6 (85.7%)	112 (68.7%)		
*Master's degree*	42 (13.5%)	7 (10.9%)	6 (17.6%)	8 (18.6%)	0 (0.0%)	21 (12.9%)		
*Middle professional education*	52 (16.7%)	6 (9.4%)	7 (20.6%)	8 (18.6%)	1 (14.3%)	30 (18.4%)	0.52	χ^2^
Marital Status								
*Married*	204 (65.6%)	38 (59.4%)	18 (52.9%)	24 (55.8%)	2 (28.6%)	122 (74.8%)		
*Not married*	67 (21.5%)	15 (23.4%)	9 (26.5%)	15 (34.9%)	4 (57.1%)	24 (14.7%)		
*Divorced/Widowed*	40 (12.9%)	11 (17.2%)	7 (20.6%)	4 (9.3%)	1 (14.3%)	17 (10.4%)	0.008	χ^2^

Classified bacterial sequencing depth varied substantially across samples, with a median of approximately 3.84 million bacterial reads per sample and a range of 357,456–14,038,630 reads. HPV-negative samples had a median classified bacterial depth of 3,671,843 reads (IQR 2,993,978–4,440,884), compared with 4,060,926 reads (IQR 3,164,986–5,175,906) in HPV-positive samples (Mann–Whitney p = 0.028). Because observed richness was positively associated with classified bacterial depth (Spearman ρ = 0.497, p < 0.001), depth-corrected sensitivity analyses were performed.

In a species-count-based depth sensitivity analysis, the higher observed richness in HPV-positive samples persisted after rarefaction of all 311 samples to an identical depth of 357,456 classified bacterial reads (median 171.5 species in HPV-positive vs 136 in HPV-negative samples; Mann–Whitney p = 1.79 × 10^–4^). The result remained significant in all 100 independent rarefaction iterations (p range 1.58 × 10^–4^–2.41 × 10^–4^; median p = 1.97 × 10^–4^). Consistently, HPV-positive status remained independently associated with higher observed richness after regression adjustment for classified bacterial depth (β = 0.313, 95% CI 0.173–0.454, p = 1.56 × 10^–5^). Accordingly, observed-richness differences and associations involving low-abundance taxa should be interpreted with consideration of effective bacterial sequencing depth. Differential-abundance analyses were conducted using relative-abundance profiles that were log-transformed within MaAsLin2, with no additional normalization applied because the input tables had already been converted to relative abundance. Bacterial sequencing depth was included as a log-transformed covariate in the adjusted models.

### 3.2 Alpha and beta diversity and taxonomic composition by HPV status

To characterize HPV-associated microbiome alterations, integrated analysis of alpha diversity, beta diversity, phylogenetic relationships, differential abundance, and taxonomic composition was performed comparing HPV-positive and HPV-negative samples. Alpha-diversity analysis at the bacterial species level showed significantly higher observed richness in HPV-positive samples than in HPV-negative samples (median 178 vs 138 species; Mann-Whitney p < 0.001). In contrast, Shannon diversity did not differ significantly between HPV-positive and HPV-negative samples (median 1.73 vs 1.49; p = 0.098), and Pielou evenness was also similar between groups (median 0.295 vs 0.294; p = 0.628). Species-level Bray-Curtis community composition did not differ significantly by HPV status (PERMANOVA: pseudo-F = 1.18, R^2^ = 0.0038, p = 0.271), and PERMDISP was not significant (p = 0.878), indicating no evidence of differential within-group dispersion (**Figure 1A**). Because observed richness was positively associated with classified bacterial sequencing depth (Spearman ρ = 0.497, p < 0.001), we performed rarefaction to the minimum classified bacterial depth of 357,456 reads per sample. The richness difference persisted after rarefaction (median 171.5 vs 136 species in HPV-positive and HPV-negative samples, respectively; Mann-Whitney p = 1.79 × 10^–4^) and remained significant in all 100 independent rarefaction iterations. In a regression model adjusting for log-transformed classified bacterial depth, HPV-positive status remained significantly associated with higher richness (β = 0.313, 95% CI 0.173–0.454, p = 1.56 × 10^–5^).

**Figure 1:**
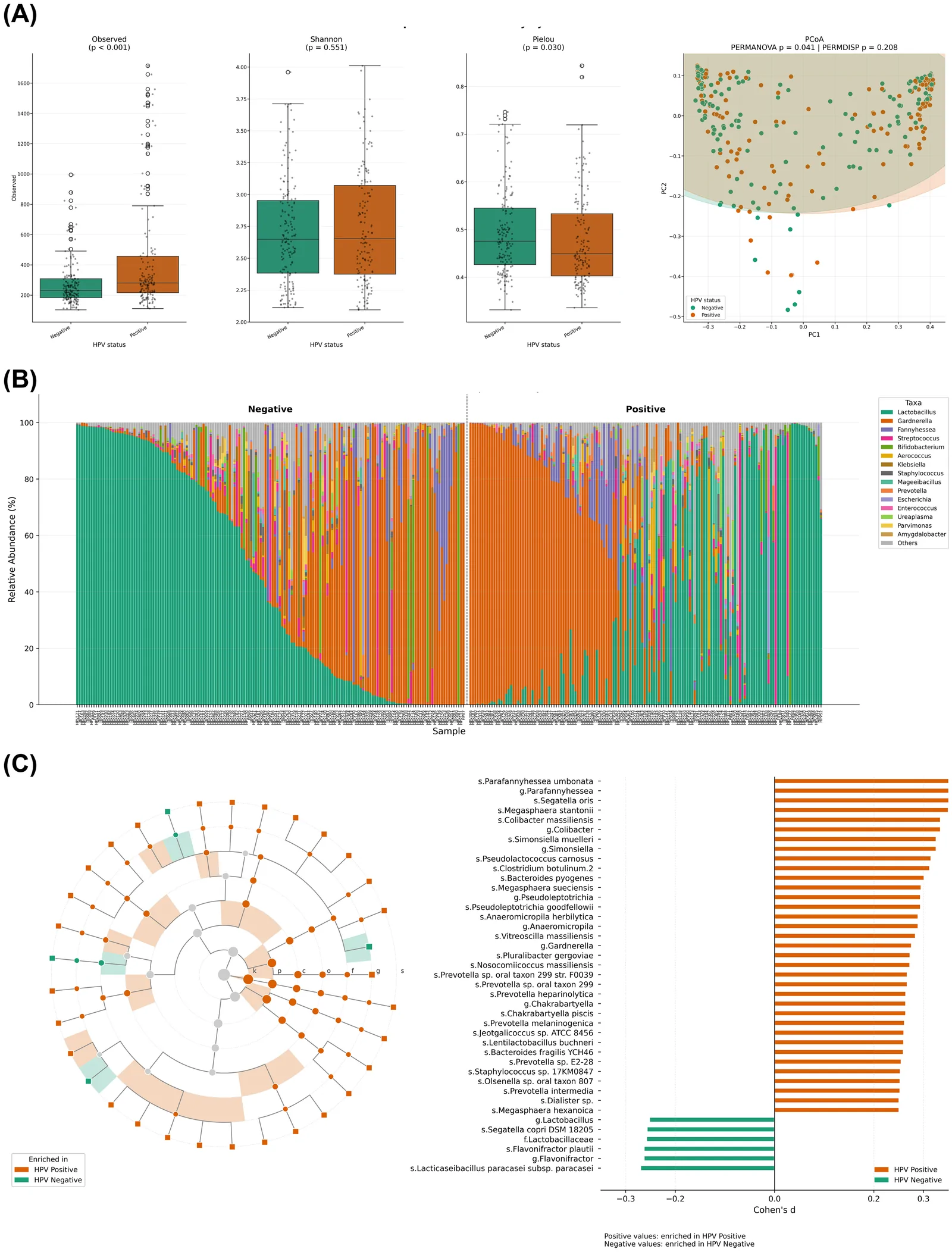
HPV status-associated variation in bacterial diversity, community composition, and differential bacterial taxa. **(A)** Bacterial alpha and beta diversity stratified by HPV status. Alpha diversity was evaluated using observed richness, Shannon diversity, and Pielou evenness, with differences between HPV-negative (n = 163) and HPV-positive (n = 148) groups assessed using the Mann–Whitney U test. Beta diversity was visualized using principal coordinates analysis based on Bray–Curtis dissimilarity, with group-level compositional differences assessed using PERMANOVA and homogeneity of dispersion evaluated using PERMDISP. **(B)** Individual-level stacked barplot showing the relative abundance of dominant bacterial genera in HPV-negative and HPV-positive samples. Each vertical bar represents one participant, and genera outside the displayed set were grouped as “Others”. **(C)** Cladogram and effect-size barplot showing bacterial taxa differing between HPV-positive and HPV-negative samples after exclusion of predefined biologically implausible and likely misclassified assignments. All taxa meeting the exploratory thresholds of p < 0.05 and |Cohen’s d> 0.25 are displayed. Positive Cohen’s d values indicate higher abundance in HPV-positive samples, whereas negative values indicate higher abundance in HPV-negative samples.

Phylum-level composition was dominated by Bacillota and Actinomycetota (>94%) in both groups. HPV-positive samples showed a reciprocal shift compared to HPV-negative — decreased Bacillota (49.4% vs 56.9%) and increased Actinomycetota (45.4% vs 37.8%), reflecting *Lactobacillus* decline and *Gardnerella* expansion. Minor phyla remained stable at <3% (**Supplementary Figure 1**). Genus-level composition showed Lactobacillus dominance in HPV-negative samples (47.6%) with Gardnerella at 29.9%, while HPV-positive samples exhibited decreased Lactobacillus (37.8%) and increased Gardnerella (41.4%), representing dominance reversal. Minor genera included Fannyhessea (5.6-6.2%), Streptococcus (3.2-4.1%), and Bifidobacterium (3.8-4.1%). The reciprocal Lactobacillus-to-Gardnerella shift reflects transition from health-associated to dysbiosis-associated profiles (**Figure 1B**).

The exploratory HPV-positive versus HPV-negative comparison identified phylogenetically diverse bacterial taxa with differences in relative abundance between the two groups after removal of predefined biologically implausible and likely misclassified assignments. HPV-positive samples showed higher abundance of taxa assigned to several anaerobic or dysbiosis-associated lineages, including *Parafannyhessea, Megasphaera, Gardnerella, Prevotella, Pseudoleptotrichia*, and related taxa. In contrast, HPV-negative samples showed higher abundance of *Flavonifractor plautii, Lacticaseibacillus paracasei, Segatella copri*, and members of the family Lactobacillaceae. The cladogram summarizes the taxonomic distribution of all taxa meeting the exploratory thresholds of p < 0.05 and |Cohen’s d> 0.25, while the accompanying barplot shows the direction and magnitude of the corresponding effect sizes (**Figure 1C**). Because this was an unadjusted exploratory comparison based on nominal p-values, these findings were interpreted as descriptive HPV-status-associated patterns rather than independently validated biomarkers. (**Figure 1C**).

### 3.3 HPV Type-specific microbiome signatures

To characterize HPV type-specific microbiome signatures, analysis was performed across No HPV, HPV Other (low-risk types), and HPV High Risk (high-risk oncogenic types). Observed species richness differed significantly across HPV type groups (Kruskal-Wallis p < 0.001), with the highest median richness in HPV Other (226 species), followed by HPV High Risk (161) and No HPV (138). Shannon diversity did not differ significantly across groups (p = 0.253), nor did Pielou evenness (p = 0.611). Species-level Bray-Curtis community composition showed only weak, non-significant separation across HPV type groups (PERMANOVA: pseudo-F = 1.50, R^2^ = 0.0096, p = 0.074), with no evidence of heterogeneous dispersion (PERMDISP p = 0.498). (**Figure 2A**).

**Figure 2:**
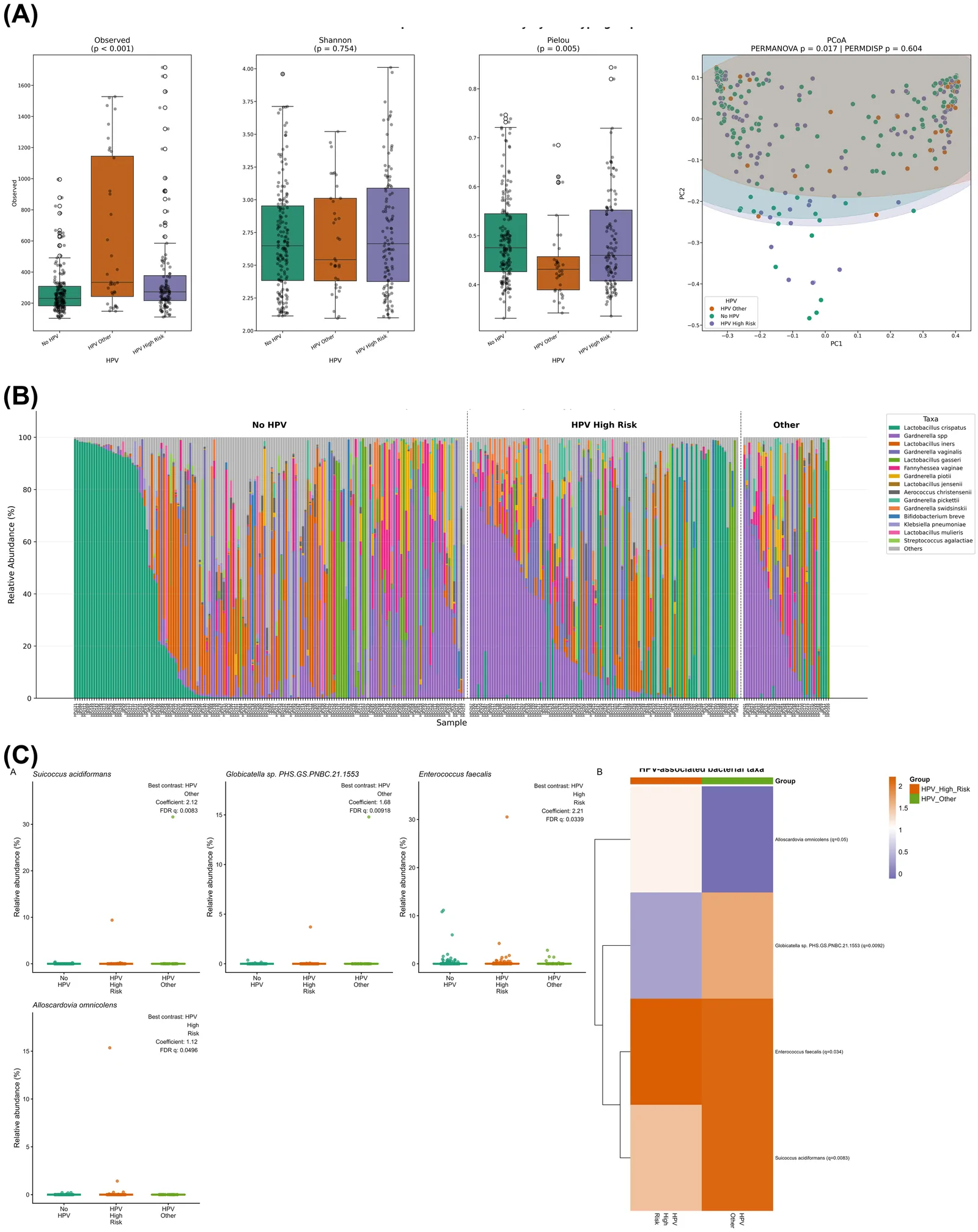
Bacterial diversity and taxonomic patterns across HPV type groups. **(A)** Bacterial alpha diversity and Bray–Curtis beta diversity across HPV type groups. The displayed groups included No HPV (n = 163), HPV Other (n = 36), and HPV High Risk, including HPV16, HPV18, and other oncogenic types (n = 112). Observed richness differed significantly among groups, while Shannon diversity and Pielou evenness were not significant; PCoA showed substantial overlap among HPV type groups, and overall species-level Bray–Curtis differences did not reach statistical significance. **(B)** Individual-level species composition revealed substantial heterogeneity and variable Lactobacillus depletion across HPV groups. **(C)** Raw relative-abundance distributions and an adjusted-coefficient heatmap for species significantly associated with HPV type group in the uncollapsed species dataset. MaAsLin2 models included HPV type group, CST, cytological grade, age, number of deliveries, log10-transformed bacterial sequencing depth, and marital status. No HPV was the reference group. Positive coefficients indicate higher abundance and negative coefficients indicate lower abundance relative to No HPV while holding the remaining covariates constant.

Genus-level composition revealed a shift between *Lactobacillus* and *Gardnerella* across HPV type groups. In No HPV and HPV High Risk samples, Lactobacillus remained dominant (44.7% and 42.6%, respectively), with Gardnerella as the second most abundant genus (28.0% and 27.1%). In contrast, HPV16, HPV18, and HPV Other groups showed a dominance reversal, with Gardnerella exceeding Lactobacillus (46.0% vs. 28.9%; 49.3% vs. 24.6%; and 46.7% vs. 30.2%, respectively), most pronounced in HPV18 and HPV16 groups (**Supplementary Figure 2**); the HPV18 pattern is based on only 7 participants and should be interpreted as exploratory.

Cervicovaginal microbiome composition stratified by HPV type revealed distinct patterns across 16 prevalent bacterial species. The No HPV group showed predominance of *Lactobacillus crispatus* with gradual transition toward mixed profiles featuring *Gardnerella* spp. and *L. iners*. HPV High Risk samples exhibited marked *Gardnerella*-dominant profiles, near-loss of *L. crispatus*, increased *Fannyhessea vaginae*, and greater inter-sample heterogeneity. The HPV Other group also displayed heterogeneous profiles with varying contributions from Lactobacillus, Gardnerella, and other anaerobic taxa. Overall, the descriptive profiles demonstrated substantial inter-individual heterogeneity and did not establish a directional transition between CSTs (**Figure 2B**).

After adjustment for CST, cytological grade, age, number of deliveries, bacterial sequencing depth, and marital status, four species were significantly associated with HPV type group (**Figure 2C**). *Suicoccus acidiformans* was positively associated with HPV Other relative to No HPV (coefficient = 2.12, q = 0.0083), and Globicatella sp. *PHS GS PNBC 21–1553* was also positively associated with HPV Other (coefficient = 1.68, q = 0.00918). Enterococcus faecalis was positively associated with HPV High Risk relative to No HPV (coefficient = 2.21, q = 0.0339), while *Alloscardovia omnicolens* showed a weaker positive association with HPV High Risk (coefficient = 1.12, q = 0.0496). These associations were heterogeneous at the individual-sample level, and several species were abundant only in subsets of participants. They should therefore be interpreted as adjusted compositional associations rather than uniformly enriched HPV-specific biomarkers.

### 3.4 Differential abundance analysis across CST and cytological grade

#### 3.4.1 CST-stratified microbiome composition and HPV effects

CST I remained *Lactobacillus crispatus*-dominant in both HPV-negative and HPV-positive samples (80.1% vs 83.2%), while CST II remained *L. gasseri*-dominant (67.8% vs 78.7%). In CST III, *L. iners* decreased from 44.2% to 36.4%. CST IV remained *Gardnerella*-rich, with *G. vaginalis* at 20.1% and 17.4%, respectively. In CST V, *L. jensenii* decreased from 55.8% to 44.4%, while *Bifidobacterium breve* increased markedly in HPV-positive samples. CST V findings should be interpreted cautiously because of the small subgroup size (**Figure 3**).

**Figure 3:**
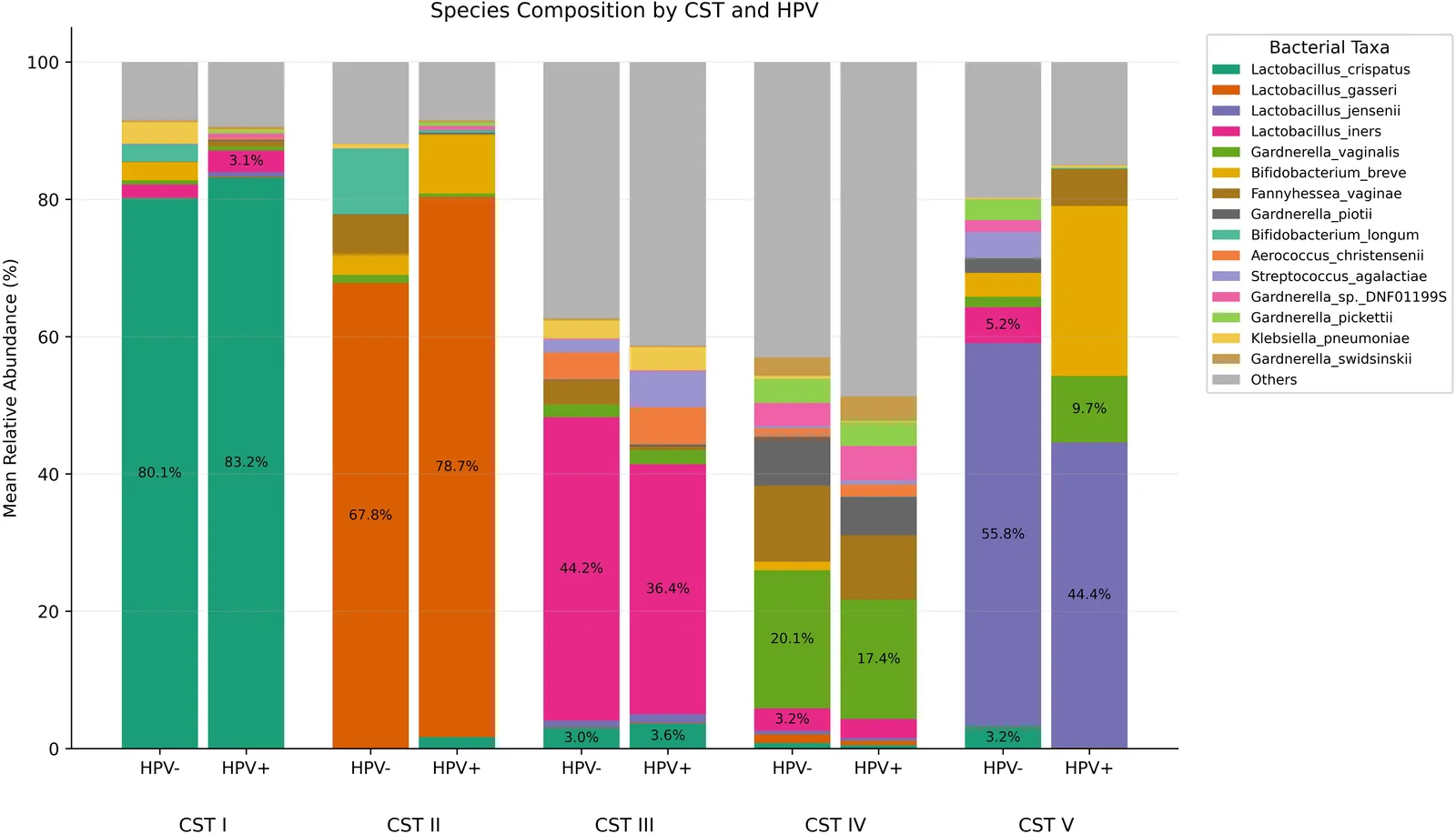
Species-level bacterial composition by community state type and HPV status. Stacked bar plots show the mean relative abundance of dominant bacterial species within CST I–V, stratified by HPV-negative and HPV-positive status. CST I, II, III, and V were defined by dominance of *Lactobacillus crispatus, L. gasseri, L. iners*, and *L. jensenii*, respectively, whereas CST IV represented a non-Lactobacillus-dominant, anaerobe-rich community. The subgroup sample sizes were CST I, HPV-negative n = 38 and HPV-positive n = 29; CST II, n = 15 and n = 10; CST III, n = 40 and n = 30; CST IV, n = 65 and n = 75; and CST V, n = 5 and n = 4, respectively. Taxa outside the displayed dominant species were grouped as “Others”. CST V findings should be interpreted cautiously because of the small subgroup sizes.

#### 3.4.2 Species composition by cytological grade and HPV status

Species-level composition stratified by cytological grade and HPV status showed substantial heterogeneity across clinical groups. In NILM samples, HPV-negative women showed higher *Lactobacillus crispatus* abundance (17.6%) than HPV-positive women (12.2%), while *Lactobacillus iners* was comparable between groups (11.4% vs. 12.0%). *Gardnerella vaginalis* was also lower in NILM HPV-positive samples (7.1%) compared with NILM HPV-negative samples (10.3%). In ASC-US, HPV-positive samples showed the highest *L. crispatus* abundance among all groups (33.1%), whereas HPV-negative ASC-US samples had lower *L. crispatus* (24.5%) but higher *L. iners* (14.6%) and *L. gasseri* (11.4%). In LSIL, HPV-positive samples showed reduced *L. crispatus* (15.5%) and increased *G. vaginalis* (12.5%) compared with HPV-negative LSIL samples (18.5% and 7.9%, respectively). ASC-H HPV-positive samples displayed the most Lactobacillus-depleted profile, with very low *L. crispatus*, modest *L. iners* (4.0%), and higher contributions from *L. gasseri* (12.7%), *Fannyhessea vaginae* (7.7%), and multiple *Gardnerella species*. The ASC-H category included only 10 participants overall (1 HPV-negative, 9 HPV-positive), and this profile should be interpreted as exploratory. Overall, these patterns suggest that HPV-associated bacterial shifts vary by cytological grade rather than following a simple linear progression (**Figure 4**).

**Figure 4:**
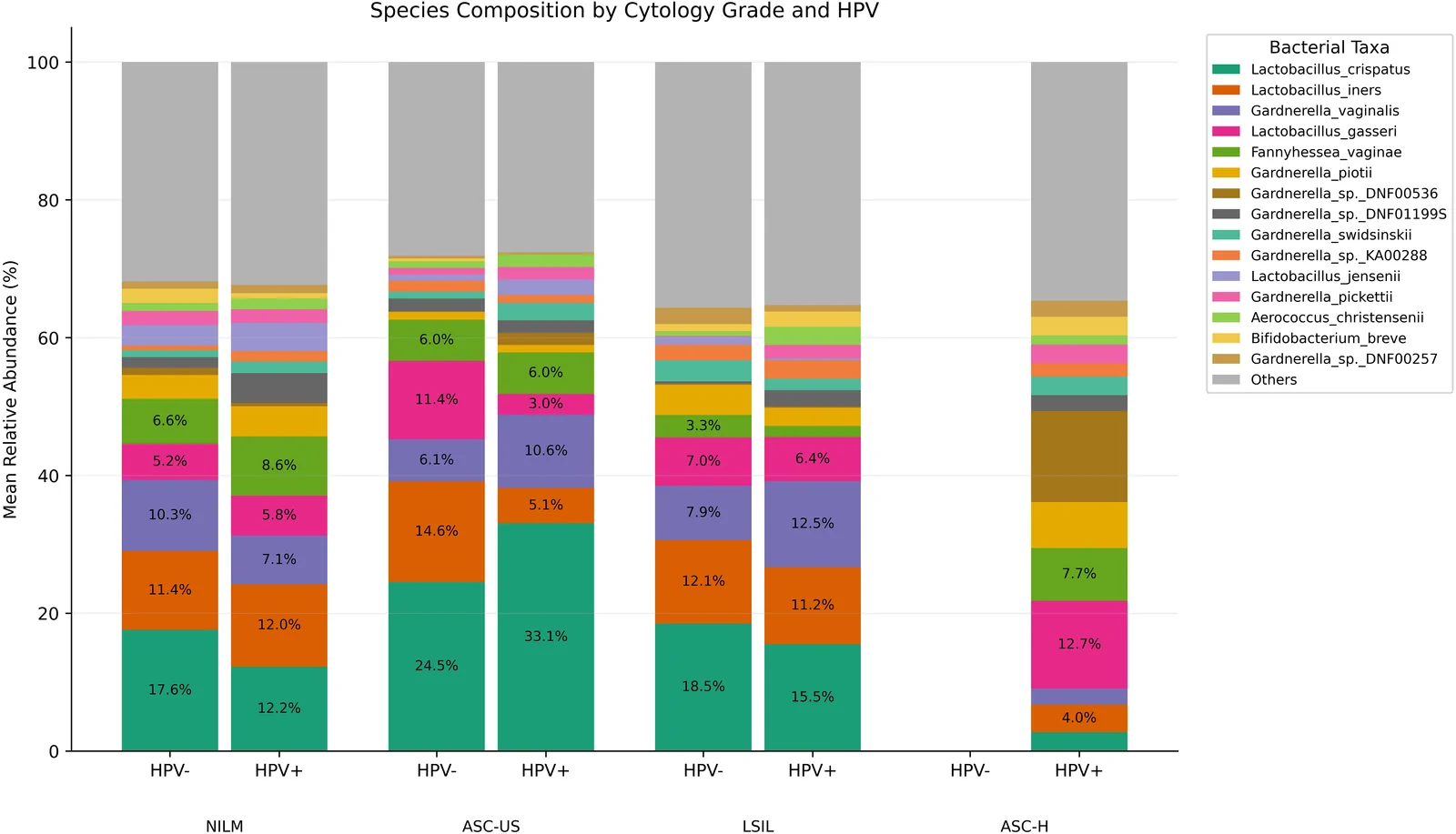
Cervical bacterial species composition across cytology-HPV subgroups. Mean relative abundance of dominant bacterial species stratified by cytological grade and HPV status. Cytological-grade information was available for 304 participants; seven participants with missing grade data were excluded from this analysis. The subgroup sample sizes were: NILM, HPV-negative n = 100 and HPV-positive n = 42; ASC-US, HPV-negative n = 39 and HPV-positive n = 34; LSIL, HPV-negative n = 20 and HPV-positive n = 59; and ASC-H, HPV-negative n = 1 and HPV-positive n = 9. Less abundant taxa were grouped as “Others”. The profiles show subgroup-specific variation in Lactobacillus- and Gardnerella-associated taxa, with the lowest Lactobacillus dominance observed in HPV-positive ASC-H samples. Findings for the ASC-H subgroup should be interpreted cautiously because it included only one HPV-negative and nine HPV-positive participants.

#### 3.4.3 CST-associated taxa

Covariate-adjusted differential-abundance analysis using the collapsed species dataset recovered the expected taxa defining the major CSTs (**Figure 5**). *Lactobacillus crispatus* was strongly negatively associated with CST IV relative to CST I (coefficient = −13.0, q = 7.48 × 10^–5^³), consistent with its predominance in CST I. *Lactobacillus gasseri* was positively associated with CST II (coefficient = 12.4, q = 1.25 × 10^–19^), whereas *Lactobacillus iners* was positively associated with CST III (coefficient = 8.47, q = 4.77 × 10^–24^). *Aerococcus christensenii* was also positively associated with CST III (coefficient = 8.94, q = 4.45 × 10^–17^). Both *Gardnerella vaginalis* and the collapsed *Gardnerella unspecified* feature were positively associated with CST IV relative to CST I. The corresponding coefficients were 7.67 for *G. vaginalis* (q = 1.51 × 10^–23^) and 8.19 for *Gardnerella unspecified* (q = 1.37 × 10^–38^). The coefficient heatmap demonstrated broad separation between Lactobacillus-dominant CSTs and the anaerobe-rich CST IV profile. Because CST assignment was based on bacterial community composition, these associations primarily confirm the internal consistency of the CST classification.

**Figure 5:**
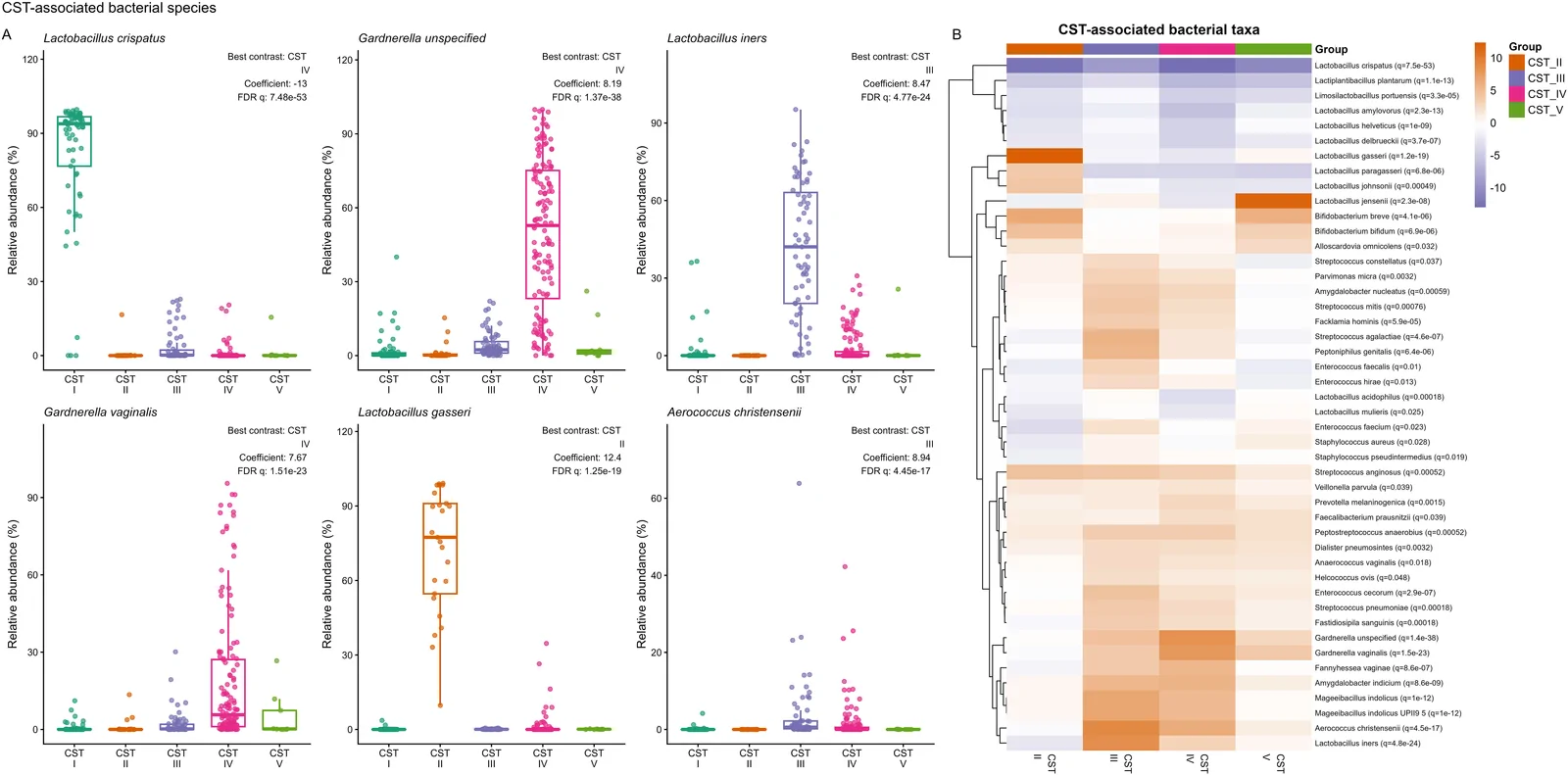
CST-associated bacterial species identified by covariate-adjusted differential-abundance analysis. **(A)** Raw relative-abundance distributions of the six species with the strongest CST associations in the collapsed species dataset. Points represent individual participants, boxes indicate the interquartile range, and horizontal lines indicate medians. MaAsLin2 models included HPV type group, CST, cytological grade, age, number of deliveries, log10-transformed bacterial sequencing depth, and marital status, with CST I as the reference category. **(B)** Heatmap of adjusted MaAsLin2 coefficients for significant CST-associated species. Positive coefficients indicate higher abundance and negative coefficients indicate lower abundance relative to CST I Gardnerella vaginalis strain assignments were collapsed as G. vaginalis, whereas other unresolved Gardnerella assignments were represented as Gardnerella unspecified.

#### 3.4.4 Cervical cytological grade-associated taxa

After adjustment for HPV type group, CST, age, number of deliveries, bacterial sequencing depth, and marital status, three species were significantly associated with cytological grade (**Figure 6**). All significant contrasts involved LSIL relative to NILM. Fannyhessea vaginae was negatively associated with LSIL (coefficient = −3.42, q = 0.0065), and Alloscardovia omnicolens was also negatively associated with LSIL (coefficient = −1.31, q = 0.0286). In contrast, Phocaeicola vulgatus was positively associated with LSIL relative to NILM (coefficient = 2.28, q = 0.0367). No species met the FDR threshold for ASC-US or ASC-H in the adjusted model. These findings support a limited LSIL-specific compositional pattern rather than a monotonic microbiome shift across increasing cytological severity.

**Figure 6:**
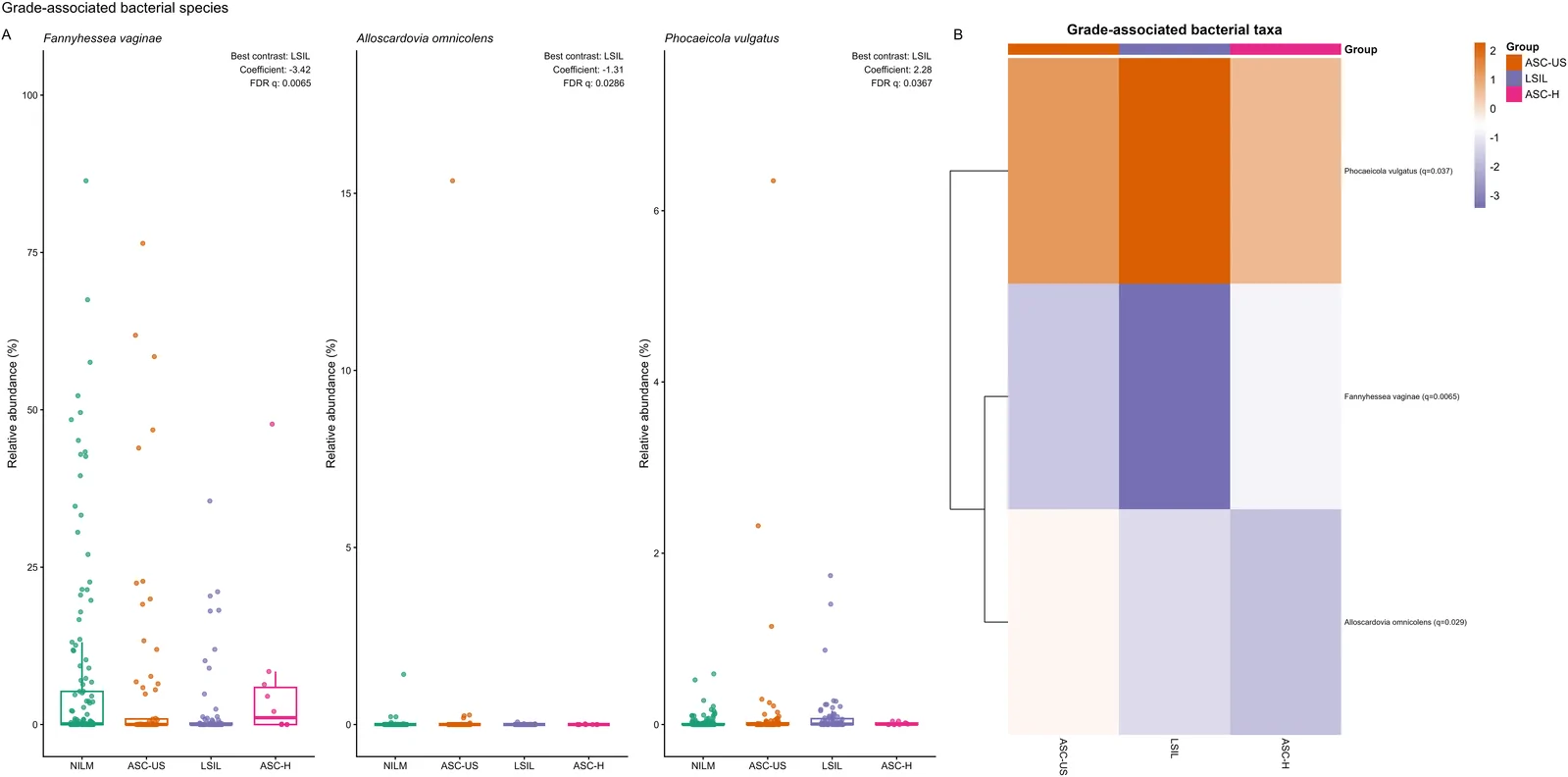
Cytological grade-associated bacterial species identified by covariate-adjusted differential-abundance analysis. **(A)** Raw relative-abundance distributions of species significantly associated with cytological grade in the uncollapsed species dataset. Points represent individual participants, boxes indicate the interquartile range, and horizontal lines indicate medians. MaAsLin2 models included HPV type group, CST, cytological grade, age, number of deliveries, log10-transformed bacterial sequencing depth, and marital status, with NILM as the reference category. **(B)** Heatmap of adjusted MaAsLin2 coefficients for significant Grade-associated species. Positive coefficients indicate higher abundance and negative coefficients indicate lower abundance relative to NILM.

In the low-depth sensitivity analysis, all three Grade-associated effects retained their direction and nominal significance, although none remained significant after FDR correction.

### 3.5 Functional pathway analysis reveals HPV type-specific metabolic alterations

Shotgun metagenomic functional profiling identified twelve significantly differential metabolic pathways across HPV type groups (**Figure 7**). HPV Other demonstrated higher predicted pathway abundance in multiple pathways including biosynthesis of siderophore group nonribosomal peptides (p = 0.0027), drug metabolism involving other enzymes (p = 0.00016), lipoic acid metabolism (p = 0.024), lipopolysaccharide biosynthesis (p = 0.0043), porphyrin metabolism (p = 0.00015), other glycan degradation (p = 0.00072), and streptomycin biosynthesis (p = 0.0011), suggesting greater predicted genetic potential and complex carbohydrate utilization characteristic of dysbiotic communities. Additionally, HPV Other showed elevated flavonoid biosynthesis (p = 0.011) and stilbenoid, diarylheptanoid and gingerol biosynthesis (p = 0.011), indicating differences in predicted pathways annotated to plant-derived compound metabolism. In contrast, No HPV samples exhibited enrichment in apoptosis of multiple species (p = 0.00025) and nucleocytoplasmic transport (p = 0.0013). Glycosphingolipid biosynthesis ganglion series pathway showed significant variation (p = 7e-06) with distinct patterns across HPV categories. HPV High Risk displayed intermediate metabolic profiles between No HPV and HPV Other for most pathways, consistent with preserved microbiome stability compared to low-risk types. These functional differences indicate that HPV type-specific microbiome alterations extend beyond taxonomic composition to encompass distinct predicted functional capacities, with low-risk HPV-associated dysbiotic communities exhibiting enhanced capacities for secondary metabolite biosynthesis, lipid metabolism, and complex glycan processing compared to health-associated microbiomes in HPV-negative or high-risk HPV-positive women.

**Figure 7:**
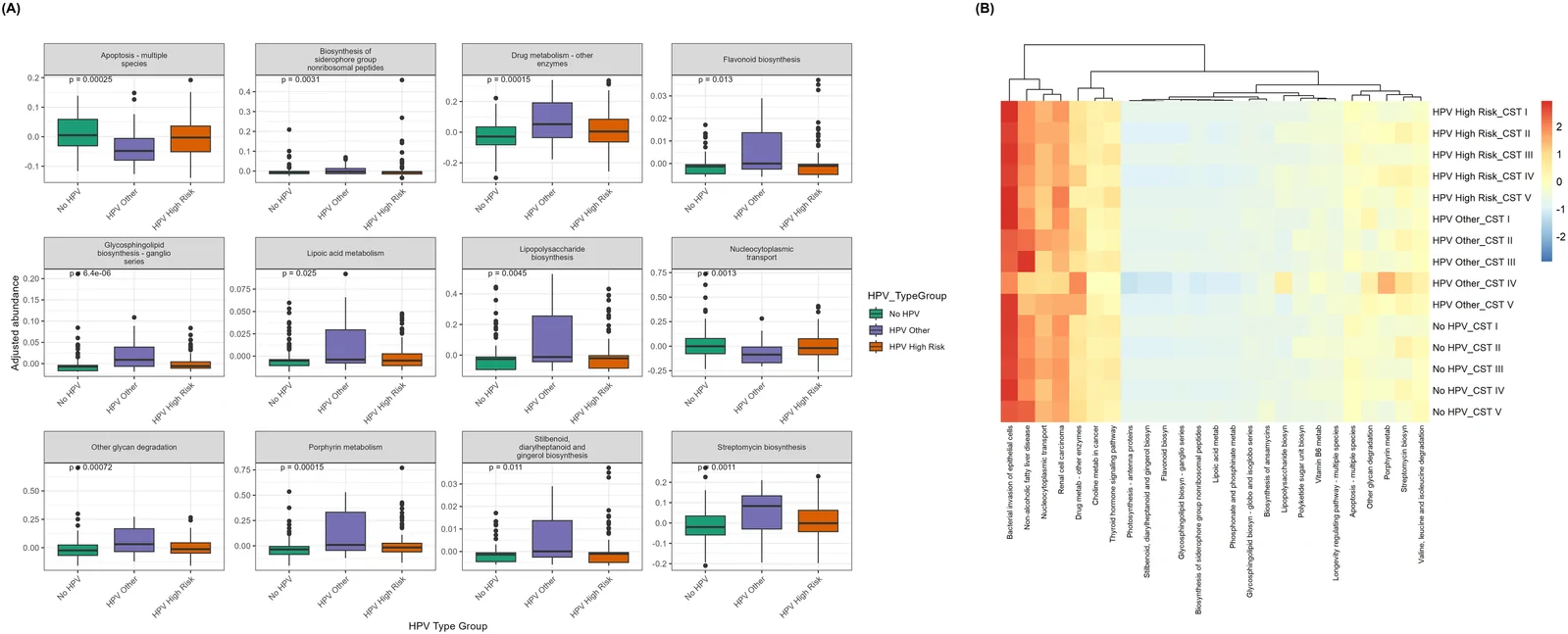
HPV type group-associated metabolic pathway variation adjusted for CST and cytological grade. **(A)** Box plots showing adjusted pathway abundances across No HPV (n = 163), HPV Other (n = 34), and HPV High Risk groups. If HPV16 and HPV18 were combined with the other oncogenic types, the HPV High Risk group included 114 participants. Boxes indicate the interquartile range, horizontal lines indicate medians, and points represent individual samples. Nominal Kruskal–Wallis p-values are shown for each pathway. **(B)** Heatmap showing pathway-abundance patterns across combined HPV type group–CST categories. Rows represent HPV type group–CST strata, and columns represent metabolic pathways. Color intensity indicates scaled pathway abundance, with hierarchical clustering applied to pathway profiles. Sample sizes for each HPV type group–CST stratum are displayed beside the corresponding row labels. HPV Other samples showed higher adjusted abundance across several pathways, including glycosphingolipid biosynthesis, lipoic acid metabolism, lipopolysaccharide biosynthesis, porphyrin metabolism, and streptomycin biosynthesis.

## 4 Discussion

In this study, we characterized cervicovaginal microbiome composition and predicted functional potential in relation to HPV status, HPV type group, cytological grade, and community state type. The HPV-negative NILM subset of the present cohort overlaps with the multi-kingdom baseline cohort described in Rakhmankulova et al. (2026); the CST distribution and Lactobacillus-dominance patterns observed here for HPV-negative women are consistent with that earlier report, supporting the internal consistency of CST assignment across the two analyses and reinforcing the present study’s characterization of HPV-positive comparator groups against this baseline. HPV-positive samples showed increased bacterial species richness, whereas Shannon diversity and Pielou evenness were not significantly different between HPV-positive and HPV-negative groups. HPV-positive samples were also enriched in phylogenetically diverse anaerobic taxa and depleted in several health-associated bacterial lineages. These findings are consistent with previous studies reporting Lactobacillus depletion and enrichment of anaerobic bacteria in HPV-positive women (Lee et al., 2013; Brotman et al., 2014; Audirac-Chalifour et al., 2016; Łaniewski et al., 2018). At the genus level, the lower relative abundance of Lactobacillus and higher abundance of Gardnerella were consistent with a shift toward a dysbiosis-associated community profile. This pattern is also consistent with meta-analyses linking bacterial vaginosis and Lactobacillus-depleted communities with HPV infection (Gillet et al., 2011; Brusselaers et al., 2019). However, the direction and mechanistic basis of this association remains debated. Dysbiosis may facilitate HPV persistence through altered mucosal immunity and reduced colonization resistance, whereas HPV-associated epithelial or inflammatory changes may themselves disrupt microbiome homeostasis (Aldunate et al., 2015; Mitra et al., 2015; Hearps et al., 2017; Shannon et al., 2017). Species-level Bray-Curtis community composition did not differ significantly by HPV status, suggesting that the principal HPV-associated diversity signal was an increase in the number of detected species rather than broad restructuring of overall community composition.

HPV Other showed the highest observed species richness, but species-level Bray-Curtis differences across HPV type groups did not reach statistical significance. This pattern differed from the expectation that greater oncogenic risk would necessarily correspond to greater microbiome alteration. However, HPV Other comprised a heterogeneous collection of genotypes, and the observed differences may reflect genotype composition, infection duration, age, reproductive characteristics, behavioral factors, or baseline microbial ecology. In the final covariate-adjusted model, HPV type group was associated with only four species. Suicoccus acidiformans and Globicatella sp. PHS GS PNBC 21–1553 were associated with HPV Other, whereas Enterococcus faecalis and Alloscardovia omnicolens were associated with HPV High Risk. The narrower set of significant taxa after adjustment suggests that some variation observed in preliminary analyses was explained by CST, grade, age, number of deliveries, sequencing depth, or marital status. Because several of these species were abundant only in subsets of participants, they should not be interpreted as stable HPV-specific biomarkers without external validation. The cross-sectional design cannot determine whether bacterial differences preceded HPV acquisition, followed infection, or reflected common underlying determinants.

Shotgun metagenomic functional profiling further suggested that HPV type-specific microbiome differences extended beyond taxonomic composition to differences in predicted microbial functional capacity. The HPV Other group showed higher inferred abundance of pathways annotated to siderophore-group nonribosomal peptide biosynthesis, drug metabolism involving other enzymes, lipoic acid metabolism, lipopolysaccharide biosynthesis, porphyrin metabolism, glycan degradation, streptomycin biosynthesis, flavonoid biosynthesis, and stilbenoid-related pathways. These findings indicate differences in the genetic potential for secondary-metabolite biosynthesis, lipid-associated processes, and complex carbohydrate degradation; however, they do not demonstrate that these pathways were transcriptionally active or that the corresponding metabolites were produced *in vivo*. Some pathways were relatively higher in HPV-negative samples, although their biological interpretation in the cervicovaginal microbiome remains uncertain. The pathway patterns were broadly consistent with the higher species richness and the taxonomic patterns observed in the HPV Other group, but they do not establish that these communities were metabolically more active or that the predicted products influenced HPV persistence or epithelial function. Metatranscriptomic and metabolomic validation will be required to determine whether these predicted pathways are expressed and whether their products are biologically relevant *in vivo*.

High-risk HPV-mediated carcinogenesis is primarily driven by viral oncogene activity, which may help explain why oncogenic HPV types were not necessarily associated with the most pronounced microbiome disruption (Zur Hausen, 2002; Doorbar et al., 2012). Conversely, the association of HPV Other with higher species richness and selected taxonomic/functional patterns may reflect differences in viral persistence, host susceptibility, or local ecological conditions rather than greater transforming potential. This pattern may reflect distinct ecological strategies: high-risk HPV types may be adapted to establish persistent infection in health-associated microbiomes where intact colonization resistance would otherwise facilitate viral clearance (Hearps et al., 2017), whereas one possibility is that different HPV categories may be associated with distinct local ecological contexts with compromised colonization resistance and altered local immunity (Molijn et al., 2005; Godoy-Vitorino et al., 2018). Alternatively, dysbiosis may impair immune-mediated clearance of low-risk types that would otherwise be rapidly eliminated, allowing their persistence without malignant transformation. This interpretation is biologically plausible because anaerobe-rich cervicovaginal communities have been associated with altered cytokine and interferon responses that may influence viral persistence across HPV types (Torcia, 2019; Mitra et al., 2020), which could theoretically facilitate viral persistence across HPV types regardless of oncogenic potential. The differing patterns observed for HPV16 and HPV18 further suggest that microbiome associations may vary among individual oncogenic genotypes (Anahtar et al., 2015; Oh et al., 2015). However, the HPV18 group included only seven participants, and its apparent microbiome pattern may therefore be strongly influenced by individual observations and should be interpreted as exploratory. Few studies have directly compared taxonomic and predicted functional profiles across HPV risk categories, because most previous investigations have focused on HPV-positive versus HPV-negative comparisons. Such binary comparisons may obscure heterogeneity among HPV genotypes and risk groups (Piyathilake et al., 2016; Mitra et al., 2017; Usyk et al., 2020), potentially obscuring important heterogeneity in HPV-microbiome associations. Rather than dysbiosis uniformly driving progression, our data suggest that HPV types differ in their associated microbiome states and metabolic activities.

CST-stratified analysis indicated that HPV-associated microbiome differences depended on the baseline community state. CST I and CST II remained predominantly Lactobacillus-dominated regardless of HPV status, suggesting greater ecological stability of these community configurations (Boris and Barbés, 2000; Tachedjian et al., 2017; Curty et al., 2020). Previous experimental studies have demonstrated that L. crispatus supernatants inhibit HPV infection *in vitro* (Boskey et al., 2001) and that Lactobacillus-dominant microbiomes associate with lower HPV viral loads *in vivo* (Kaelin et al., 2022), supporting protective roles of these bacteria against viral persistence. CST III, characterized by L. iners dominance, demonstrated moderate vulnerability to HPV-associated dysbiosis with notable expansion of opportunistic pathogens including Klebsiella. This intermediate susceptibility may reflect L. iners’ distinct physiology compared to other Lactobacillus species. Unlike L. crispatus and L. gasseri, L. iners produces minimal lactic acid through limited D-lactate production, lacks capacity for hydrogen peroxide generation, and exhibits smaller genome size with reduced metabolic versatility (Guijon et al., 1992; France et al., 2020). Longitudinal studies have similarly shown that L. iners-dominated communities are more likely than L. crispatus-dominated communities to transition toward CST IV (Macklaim et al., 2013; Ravel et al., 2013), consistent with our observations of intermediate vulnerability. CST IV and CST V showed pronounced HPV-associated compositional differences. CST IV exhibited near-complete Lactobacillus collapse with further Gardnerella expansion, indicating that baseline dysbiosis amplifies HPV-associated ecological destabilization. This finding is consistent with previous reports associating bacterial vaginosis and CST IV with HPV acquisition, persistence, and cervical abnormalities (Watts et al., 2005; Jespers et al., 2012; Champer et al., 2018; Sadeghpour Heravi, 2024). CST V showed reduced Lactobacillus and increased Bifidobacterium abundance among HPV-positive samples, although this pattern should be interpreted cautiously because CST V included few participants. Overall, the findings suggest that baseline CST modifies the association between HPV status and cervicovaginal microbiome composition.

Species-level analysis revealed distinct, but not strictly progressive, microbiome patterns across cytological grades. The adjusted Grade analysis identified a limited LSIL-associated pattern. Fannyhessea vaginae and Alloscardovia omnicolens were less abundant in LSIL than in NILM, whereas Phocaeicola vulgatus was more abundant. No species remained significantly associated with ASC-US or ASC-H after multivariable adjustment. *Fannyhessea vaginae* (formerly *Atopobium vaginae*) has been implicated in bacterial vaginosis (Zeng et al., 2023) but may play protective roles in certain contexts, and its depletion in LSIL warrants further investigation. The relatively high abundance of *L. crispatus* in HPV-positive ASC-US samples further indicates that microbiome composition does not change monotonically with cytological severity. The severe dysbiosis observed in ASC-H samples with near-complete *L. crispatus* loss and substantial Gardnerella strain diversification suggests that high-grade cytological abnormalities associate with profound compositional restructuring. Previous studies have reported increased microbial diversity and *Lactobacillus depletion* in cervical intraepithelial neoplasia and cervical cancer (Li et al., 2020; Onywera et al., 2020; Hu et al., 2022), consistent with our observations. However, the direction of causality remains unclear: dysbiosis may promote disease progression through inflammation and immune dysfunction (Klein et al., 2019; Pęczek et al., 2023), or alternatively, epithelial transformation and tissue remodeling may secondarily disrupt microbiome homeostasis (Fernandes et al., 2024). The presence of intestinal taxa including *Bacteroides* species in high-grade lesions raises questions about fecal contamination, sexual practices facilitating bacterial translocation, or true colonization of the cervicovaginal niche by enteric bacteria in the context of epithelial disruption. Interpretation of ASC-H-specific patterns is also limited by the small number of participants in this cytological category.

Several potential mechanisms may underlie the observed HPV-microbiome associations. First, microbiome composition may influence HPV persistence through modulation of local immunity. Lactobacillus-dominant communities have been associated with epithelial integrity and balanced mucosal immune responses, whereas anaerobe-rich communities may promote inflammation and barrier disruption that could impair viral clearance (Adurthi et al., 2017; Eastment and McClelland, 2018; Leon-Gomez and Romero, 2024; Alizhan et al., 2025). Second, bacterial metabolites may directly affect HPV infection or replication. The observed enrichment of secondary metabolite biosynthesis pathways in low-risk HPV-associated dysbiotic communities suggests potential roles for bacterial products in modulating viral-host interactions. Short-chain fatty acids produced by certain anaerobes can modulate histone acetylation and gene expression (Łaniewski et al., 2019), potentially influencing HPV oncogene expression or host antiviral responses. Siderophores, lipopolysaccharides, and other bacterial metabolites enriched in dysbiotic environments may similarly affect epithelial cell signaling, immune responses, or viral replication. Third, microbiome-mediated alterations in vaginal pH may influence HPV infection efficiency or viral shedding. Lactic acid production by Lactobacillus species maintains acidic pH that can inhibit certain pathogens (Hou et al., 2024), though direct effects on HPV are incompletely characterized. Dysbiosis-associated bacterial enzymes and virulence factors may also weaken mucus and epithelial barriers, potentially increasing susceptibility to infection (Liu and Li, 2024; Papamentzelopoulou and Pitiriga, 2025). Finally, microbiome composition may serve as a biomarker of underlying immune or hormonal status that independently influences both microbiome state and HPV susceptibility, representing correlation without direct causation.

Several limitations warrant consideration. First, the cross-sectional design precludes determination of temporal relationships and causality-whether HPV infection drives microbiome alterations, dysbiosis facilitates HPV persistence, or bidirectional interactions occur. Longitudinal studies tracking microbiome composition from HPV acquisition through viral clearance or persistence and disease progression are needed to resolve these questions and identify predictive biomarkers. Second, lack of detailed data on sexual behavior, contraceptive use, menstrual cycle phase, recent antibiotic exposure, and other potential confounders limits ability to adjust for alternative explanations of observed associations. Third, several subgroup analyses were limited by small sample sizes. Fourth, single time-point sampling cannot account for temporal variability in microbiome composition, which previous studies have shown can fluctuate across menstrual cycles or in response to sexual activity (Noyes et al., 2018; Brockhausen et al., 2024).

An additional limitation is the substantial sequencing inefficiency caused by the high host-DNA content of cervicovaginal samples. Although approximately 48 million raw reads were generated per sample, the median bacterial depth was only ~3.8 million reads. Variable microbial depth may reduce sensitivity for low-abundance taxa and contribute to compositional distortion, and no prespecified minimum non-host depth threshold was applied. Nevertheless, depth-adjusted analyses and exclusion of the lowest-depth quartile showed that the principal associations remained directionally consistent. Future studies should incorporate predefined microbial-depth thresholds and consider reduced-representation approaches such as 2bRAD-M, which can profile host-rich samples without prior host depletion and with substantially lower sequencing requirements than whole-metagenome sequencing (Song et al., 2020). However, its performance should be specifically validated in cervicovaginal samples. Finally, HUMAnN pathway results represent predicted functional potential rather than measured gene expression or metabolite production. Classified bacterial depth varied substantially among samples and was associated with observed richness. Importantly, the HPV-associated richness difference persisted after rarefaction to identical bacterial depth, and MaAsLin2 effect estimates were highly concordant after exclusion of the lowest-depth quartile. The three Grade-associated effects retained direction and nominal significance after exclusion of the lowest-depth quartile but no longer met the FDR threshold, indicating that these LSIL-associated findings should be regarded as hypothesis-generating and require validation in larger cohorts. HPV-associated coefficient directions were also preserved after low-depth exclusion, although only two of five contrasts retained FDR significance, suggesting that some individual taxon-level associations have limited statistical stability after reduction in sample size. Future studies should combine longitudinal sampling with genotype-specific HPV measurements, viral-load assessment, metatranscriptomics, metabolomics, and host immune profiling to validate the taxonomic and predicted functional associations observed here. Interventional studies will ultimately be required to determine whether microbiome modification influences HPV clearance or lesion outcomes (Palma et al., 2018; Ou et al., 2019). Experimental studies of specific taxa and microbial products would also help test mechanisms suggested by the metagenomic findings. Strain-resolved analyses, including metagenome-assembled genomes and virulence-gene profiling, may further clarify functional variation among dominant cervicovaginal taxa.

The observed associations raise the possibility that microbiome features, including CST and predicted functional profiles, may eventually complement established HPV genotype- and cytology-based assessment. However, the present cross-sectional data do not establish that particular CSTs predict HPV clearance, persistence, or lesion progression. Importantly, the greater dysbiosis observed in the HPV Other group indicates that microbiome disruption should not be interpreted as a direct proxy for oncogenic risk. Microbiome differences may instead reflect distinct HPV–host ecological contexts, shared risk factors, or consequences of infection and tissue change. Prospective studies are needed to determine whether microbiome features provide predictive value beyond established clinical markers and whether any microbiome-targeted approach can safely influence HPV persistence or lesion outcomes (Kozhakhmetov et al., 2023; Onyango et al., 2023; Pedro and Mira, 2024; Jiang et al., 2025; Nazarova et al., 2026).

## 5 Conclusions

This study demonstrates that HPV infection, HPV type group, cytological grade, and community state type are associated with distinct cervicovaginal microbiome patterns. HPV-positive women showed greater bacterial species richness and shifts in dominant bacterial taxa, whereas Shannon diversity, Pielou evenness, and overall species-level Bray–Curtis composition did not differ significantly by HPV status. CST analyses confirmed the expected separation between Lactobacillus-dominant and anaerobe-rich communities. Predicted functional differences were also observed across HPV groups, but these findings represent microbial potential rather than measured activity. Overall, the results support a complex relationship between HPV and the cervicovaginal microbiome, while the cross-sectional design prevents conclusions about causality or disease progression.

## Data Availability

The datasets presented in this study can be found in online repositories. The names of the repository/repositories and accession number(s) can be found below: https://www.ncbi.nlm.nih.gov/, PRJNA1337009.
