# Supplementary material for: Cervicovaginal microbiome alterations across HPV infection
Source: Front Cell Infect Microbiol. 2026 Sep 21;16:1907605. doi: 10.3389/fcimb.2026.1907605 (PMC13634844; doi:10.3389/fcimb.2026.1907605)
Supplement: Supplementary file 1 [file Table1.docx]

Supplementary Table 1. Complete MaAsLin2 differential-abundance results for HPV type groups

| feature | metadata | value | coef | stderr | N | N.not.0 | pval | qval |
| --- | --- | --- | --- | --- | --- | --- | --- | --- |
| s_Suicoccus_acidiformans | HPV_TypeGroup | HPV_Other | 2,12318 | 0,61480 | 302 | 52 | 0,00064 | 0,00830 |
| s_Globicatella_sp._  PHS.GS.PNBC.21.1553 | HPV_TypeGroup | HPV_Other | 1,67715 | 0,49074 | 302 | 37 | 0,00072 | 0,00918 |
| s_Suicoccus_acidiformans | HPV_TypeGroup | HPV_High_Risk | 1,49929 | 0,47281 | 302 | 52 | 0,00168 | 0,01911 |
| s_Enterococcus_faecalis | HPV_TypeGroup | HPV_High_Risk | 2,21310 | 0,74690 | 302 | 198 | 0,00330 | 0,03387 |
| s_Alloscardovia_omnicolens | HPV_TypeGroup | HPV_High_Risk | 1,12431 | 0,39925 | 302 | 49 | 0,00520 | 0,04960 |

Supplementary Table 2. Complete MaAsLin2 differential-abundance results for Cytology Grade groups.

| feature | metadata | value | coef | stderr | N | N,not,0 | pval | qval |
| --- | --- | --- | --- | --- | --- | --- | --- | --- |
| s_Fannyhessea_vaginae | Grade | LSIL | -3,4236 | 0,9675 | 302 | 199 | 0,0005 | 0,0065 |
| s_Alloscardovia_omnicolens | Grade | LSIL | -1,3094 | 0,4322 | 302 | 49 | 0,0027 | 0,0286 |
| s_Phocaeicola_vulgatus | Grade | LSIL | 2,2760 | 0,7755 | 302 | 175 | 0,0036 | 0,0367 |

Supplementary Table 3. Complete MaAsLin2 differential-abundance results for CST groups.

| feature | metadata | value | coef | stderr | N | N,not,0 | pval | qval |
| --- | --- | --- | --- | --- | --- | --- | --- | --- |
| s_Lactobacillus_crispatus | CST_group | CST_IV | -13,025 | 0,656 | 302 | 283 | 7,32E-56 | 7,48E-53 |
| s_Gardnerella_unspecified | CST_group | CST_IV | 8,188 | 0,516 | 302 | 277 | 4,02E-41 | 1,37E-38 |
| s_Lactobacillus_crispatus | CST_group | CST_II | -12,299 | 0,983 | 302 | 283 | 5,90E-29 | 1,21E-26 |
| s_Lactobacillus_iners | CST_group | CST_III | 8,470 | 0,721 | 302 | 291 | 2,80E-26 | 4,77E-24 |
| s_Lactobacillus_crispatus | CST_group | CST_III | -9,012 | 0,772 | 302 | 283 | 5,02E-26 | 7,33E-24 |
| s_Gardnerella_vaginalis | CST_group | CST_IV | 7,670 | 0,663 | 302 | 246 | 1,18E-25 | 1,51E-23 |
| s_Lactobacillus_gasseri | CST_group | CST_II | 12,370 | 1,190 | 302 | 238 | 1,10E-21 | 1,25E-19 |
| s_Aerococcus_christensenii | CST_group | CST_III | 8,939 | 0,934 | 302 | 208 | 5,23E-19 | 4,45E-17 |
| s_Lactiplantibacillus_plantarum | CST_group | CST_IV | -6,613 | 0,782 | 302 | 205 | 1,45E-15 | 1,06E-13 |
| s_Lactobacillus_amylovorus | CST_group | CST_IV | -5,807 | 0,697 | 302 | 214 | 3,31E-15 | 2,26E-13 |
| s_Mageeibacillus_indolicus_  UPII9_5 | CST_group | CST_III | 6,760 | 0,836 | 302 | 171 | 1,70E-14 | 1,02E-12 |
| s_Mageeibacillus_indolicus | CST_group | CST_III | 6,760 | 0,836 | 302 | 171 | 1,70E-14 | 1,02E-12 |
| s_Aerococcus_christensenii | CST_group | CST_IV | 6,169 | 0,793 | 302 | 208 | 1,34E-13 | 7,62E-12 |
| s_Gardnerella_unspecified | CST_group | CST_III | 4,715 | 0,608 | 302 | 277 | 1,54E-13 | 8,30E-12 |
| s_Lactobacillus_crispatus | CST_group | CST_V | -10,682 | 1,491 | 302 | 283 | 6,60E-12 | 3,37E-10 |
| s_Mageeibacillus_indolicus_  UPII9_5 | CST_group | CST_IV | 5,048 | 0,709 | 302 | 171 | 8,95E-12 | 4,16E-10 |
| s_Mageeibacillus_indolicus | CST_group | CST_IV | 5,048 | 0,709 | 302 | 171 | 8,95E-12 | 4,16E-10 |
| s_Lactobacillus_helveticus | CST_group | CST_IV | -4,291 | 0,617 | 302 | 248 | 2,32E-11 | 1,03E-09 |
| s_Amygdalobacter_indicium | CST_group | CST_IV | 5,627 | 0,853 | 302 | 179 | 2,02E-10 | 8,61E-09 |
| s_Lactobacillus_jensenii | CST_group | CST_V | 11,704 | 1,824 | 302 | 217 | 5,72E-10 | 2,34E-08 |
| s_Enterococcus_cecorum | CST_group | CST_III | 4,490 | 0,753 | 302 | 128 | 7,26E-09 | 2,85E-07 |
| s_Lactobacillus_delbrueckii | CST_group | CST_IV | -4,220 | 0,714 | 302 | 196 | 9,76E-09 | 3,69E-07 |
| s_Streptococcus_agalactiae | CST_group | CST_III | 5,622 | 0,959 | 302 | 207 | 1,26E-08 | 4,60E-07 |
| s_Fannyhessea_vaginae | CST_group | CST_IV | 5,400 | 0,941 | 302 | 199 | 2,44E-08 | 8,60E-07 |
| s_Amygdalobacter_indicium | CST_group | CST_III | 5,463 | 1,005 | 302 | 179 | 1,15E-07 | 3,93E-06 |
| s_Bifidobacterium_breve | CST_group | CST_II | 6,357 | 1,172 | 302 | 93 | 1,24E-07 | 4,10E-06 |
| s_Peptoniphilus_genitalis | CST_group | CST_III | 5,402 | 1,014 | 302 | 180 | 2,00E-07 | 6,37E-06 |
| s_Lactobacillus_paragasseri | CST_group | CST_IV | -4,058 | 0,764 | 302 | 180 | 2,21E-07 | 6,83E-06 |
| s_Bifidobacterium_bifidum | CST_group | CST_II | 4,679 | 0,882 | 302 | 60 | 2,28E-07 | 6,86E-06 |
| s_Lactobacillus_iners | CST_group | CST_IV | 3,217 | 0,612 | 302 | 291 | 2,91E-07 | 8,49E-06 |
| s_Limosilactobacillus_portuensis | CST_group | CST_IV | -4,636 | 0,935 | 302 | 130 | 1,22E-06 | 3,29E-05 |
| s_Gardnerella_vaginalis | CST_group | CST_III | 3,798 | 0,781 | 302 | 246 | 1,90E-06 | 4,99E-05 |
| s_Facklamia_hominis | CST_group | CST_III | 3,569 | 0,740 | 302 | 113 | 2,32E-06 | 5,94E-05 |
| s_Lactobacillus_paragasseri | CST_group | CST_III | -4,133 | 0,900 | 302 | 180 | 6,59E-06 | 1,64E-04 |
| s_Streptococcus_pneumoniae | CST_group | CST_III | 3,913 | 0,857 | 302 | 178 | 7,36E-06 | 1,79E-04 |
| s_Fastidiosipila_sanguinis | CST_group | CST_III | 3,595 | 0,789 | 302 | 98 | 7,75E-06 | 1,80E-04 |
| s_Lactobacillus_acidophilus | CST_group | CST_IV | -3,133 | 0,687 | 302 | 213 | 7,60E-06 | 1,80E-04 |
| s_Lactiplantibacillus_plantarum | CST_group | CST_II | -5,091 | 1,173 | 302 | 205 | 1,98E-05 | 4,40E-04 |
| s_Lactobacillus_johnsonii | CST_group | CST_II | 4,017 | 0,932 | 302 | 247 | 2,24E-05 | 4,88E-04 |
| s_Peptostreptococcus_anaerobius | CST_group | CST_IV | 3,627 | 0,846 | 302 | 138 | 2,48E-05 | 5,17E-04 |
| s_Streptococcus_anginosus | CST_group | CST_III | 4,143 | 0,968 | 302 | 177 | 2,55E-05 | 5,22E-04 |
| s_Amygdalobacter_nucleatus | CST_group | CST_III | 3,917 | 0,922 | 302 | 124 | 2,94E-05 | 5,89E-04 |
| s_Lactobacillus_johnsonii | CST_group | CST_IV | -2,618 | 0,621 | 302 | 247 | 3,38E-05 | 6,52E-04 |
| s_Streptococcus_mitis | CST_group | CST_III | 4,165 | 0,999 | 302 | 144 | 4,04E-05 | 7,65E-04 |
| s_Streptococcus_anginosus | CST_group | CST_IV | 3,294 | 0,822 | 302 | 177 | 7,82E-05 | 1,45E-03 |
| s_Prevotella_melaninogenica | CST_group | CST_IV | 2,936 | 0,736 | 302 | 124 | 8,43E-05 | 1,54E-03 |
| s_Lactiplantibacillus_plantarum | CST_group | CST_III | -3,615 | 0,921 | 302 | 205 | 1,09E-04 | 1,96E-03 |
| s_Parvimonas_micra | CST_group | CST_III | 3,057 | 0,808 | 302 | 109 | 1,86E-04 | 3,17E-03 |
| s_Dialister_pneumosintes | CST_group | CST_IV | 2,432 | 0,642 | 302 | 92 | 1,86E-04 | 3,17E-03 |
| s_Streptococcus_pneumoniae | CST_group | CST_IV | 2,642 | 0,728 | 302 | 178 | 3,34E-04 | 5,51E-03 |
| s_Peptostreptococcus_anaerobius | CST_group | CST_III | 3,606 | 0,996 | 302 | 138 | 3,50E-04 | 5,67E-03 |
| s_Fannyhessea_vaginae | CST_group | CST_III | 3,910 | 1,108 | 302 | 199 | 4,89E-04 | 7,57E-03 |
| s_Amygdalobacter_nucleatus | CST_group | CST_IV | 2,749 | 0,783 | 302 | 124 | 5,21E-04 | 7,95E-03 |
| s_Streptococcus_anginosus | CST_group | CST_II | 4,257 | 1,233 | 302 | 177 | 6,36E-04 | 9,43E-03 |
| s_Lactobacillus_paragasseri | CST_group | CST_II | 3,947 | 1,146 | 302 | 180 | 6,60E-04 | 9,64E-03 |
| s_Enterococcus_faecalis | CST_group | CST_III | 3,155 | 0,926 | 302 | 198 | 7,53E-04 | 1,04E-02 |
| s_Fastidiosipila_sanguinis | CST_group | CST_IV | 2,281 | 0,670 | 302 | 98 | 7,58E-04 | 1,04E-02 |
| s_Dialister_pneumosintes | CST_group | CST_III | 2,538 | 0,757 | 302 | 92 | 9,01E-04 | 1,20E-02 |
| s_Bifidobacterium_breve | CST_group | CST_V | 5,945 | 1,778 | 302 | 93 | 9,35E-04 | 1,22E-02 |
| s_Facklamia_hominis | CST_group | CST_IV | 2,100 | 0,629 | 302 | 113 | 9,50E-04 | 1,23E-02 |
| s_Enterococcus_hirae | CST_group | CST_III | 2,523 | 0,761 | 302 | 83 | 1,02E-03 | 1,31E-02 |
| s_Enterococcus_cecorum | CST_group | CST_IV | 2,067 | 0,639 | 302 | 128 | 1,37E-03 | 1,70E-02 |
| s_Anaerococcus_vaginalis | CST_group | CST_III | 2,704 | 0,840 | 302 | 119 | 1,44E-03 | 1,77E-02 |
| s_Staphylococcus_  pseudintermedius | CST_group | CST_II | -1,654 | 0,518 | 302 | 293 | 1,56E-03 | 1,90E-02 |
| s_Lactobacillus_jensenii | CST_group | CST_IV | -2,557 | 0,802 | 302 | 217 | 1,59E-03 | 1,91E-02 |
| s_Enterococcus_faecium | CST_group | CST_II | -3,453 | 1,103 | 302 | 175 | 1,93E-03 | 2,27E-02 |
| s_Lactobacillus_gasseri | CST_group | CST_IV | -2,466 | 0,794 | 302 | 238 | 2,08E-03 | 2,38E-02 |
| s_Lactiplantibacillus_plantarum | CST_group | CST_V | -5,515 | 1,779 | 302 | 205 | 2,13E-03 | 2,42E-02 |
| s_Lactobacillus_amylovorus | CST_group | CST_II | -3,230 | 1,045 | 302 | 214 | 2,20E-03 | 2,47E-02 |
| s_Lactobacillus_mulieris | CST_group | CST_IV | -2,529 | 0,822 | 302 | 198 | 2,28E-03 | 2,53E-02 |
| s_Lactobacillus_helveticus | CST_group | CST_II | -2,834 | 0,925 | 302 | 248 | 2,38E-03 | 2,62E-02 |
| s_Staphylococcus_aureus | CST_group | CST_II | -2,274 | 0,749 | 302 | 282 | 2,62E-03 | 2,85E-02 |
| s_Alloscardovia_omnicolens | CST_group | CST_II | 1,883 | 0,631 | 302 | 49 | 3,07E-03 | 3,24E-02 |
| s_Streptococcus_constellatus | CST_group | CST_III | 2,503 | 0,854 | 302 | 91 | 3,64E-03 | 3,68E-02 |
| s_Parvimonas_micra | CST_group | CST_IV | 1,996 | 0,686 | 302 | 109 | 3,89E-03 | 3,86E-02 |
| s_Veillonella_parvula | CST_group | CST_IV | 1,936 | 0,665 | 302 | 99 | 3,87E-03 | 3,86E-02 |
| s_Faecalibacterium_prausnitzii | CST_group | CST_IV | 2,299 | 0,794 | 302 | 147 | 4,05E-03 | 3,94E-02 |
| s_Helcococcus_ovis | CST_group | CST_III | 2,394 | 0,846 | 302 | 106 | 4,98E-03 | 4,81E-02 |
